# Individual and combined association analysis of famine exposure and serum uric acid with hypertension in the mid-aged and older adult: a population-based cross-sectional study

**DOI:** 10.1186/s12872-021-02230-z

**Published:** 2021-09-06

**Authors:** Lin Zhang, Liu Yang, Congzhi Wang, Ting Yuan, Dongmei Zhang, Huanhuan Wei, Jing Li, Yunxiao Lei, Lu Sun, Xiaoping Li, Ying Hua, Hengying Che, Yuanzhen Li

**Affiliations:** 1 Department of Internal Medicine Nursing, School of Nursing, Wannan Medical College, 22 Wenchang West Road, Higher Education Park, Wuhu City, An Hui Province People’s Republic of China; 2 Obstetrics and Gynecology Nursing, School of Nursing, Wannan Medical College, 22 Wenchang West Road, Higher Education Park, Wuhu City, An Hui Province People’s Republic of China; 3 Department of Pediatric Nursing, School of Nursing, Wannan Medical College, 22 Wenchang West Road, Higher Education Park, Wuhu City, An Hui Province People’s Republic of China; 4 Department of Surgical Nursing, School of Nursing, Wannan Medical College, 22 Wenchang West Road, Higher Education Park, Wuhu City, An Hui Province People’s Republic of China; 5 Department of Emergency and Critical Care Nursing, School of Nursing, Wannan Medical College, 22 Wenchang West Road, Higher Education Park, Wuhu City, An Hui Province People’s Republic of China; 6 Rehabilitation Nursing, School of Nursing, Wannan Medical College, 22 Wenchang West Road, Higher Education Park, Wuhu City, An Hui Province People’s Republic of China; 7 Department of Nursing, Yijishan Hospital, The First Affiliated Hospital of Wannan Medical College, Zheshan West Road, Yijishan District, Wuhu City, Anhui Province People’s Republic of China

**Keywords:** Body mass index, Famine exposure, Hypertension, Serum uric acid, Individual and combined association

## Abstract

**Background:**

Malnutrition in early life may affect health in later life. The associations between malnutrition and serum uric acid (SUA) and hypertension were inconsistent. The present study aimed to investigate the individual and combined association between famine exposure and serum uric acid and hypertension in middle-aged and older Chinese.

**Methods:**

Data were selected from the China Health and Retirement Longitudinal Study (CHARLS) Wave2011. The analytic sample included 9368 individuals aged 45 to 90. Differences between baseline characteristics and famine exposure/SUA level were evaluated using the Chi-square test, t-test, and F-test. Then, the differences in the prevalence of hypertension between characteristic groups was also estimated by the Chi-square and t-test. Finally, multivariable-adjusted logistic regression models examined association of famine exposure and serum uric acid with odds of prevalence of hypertension.

**Results:**

A total of 9368 individuals were enrolled in the study, 4366 (46.61%) and 5002 (53.39%) were male and female, respectively. Among males, 459 (10.51%) had been exposed to the Chinese famine during the fetal stage, whereas 1760 (40.31%) and 1645 (37.68%) had been exposed to the famine during childhood and adolescence/adult stage, respectively. Among females, 635 (12.69%) had been exposed to the Chinese famine during the fetal stage, whereas 1988 (39.74%) and 1569 (31.37%) had been exposed to the famine during childhood and adolescence/adult stage, respectively. Regarding the participants with SUA level measurements, 290 (6.64%) reported having Hyperuricemia (HUA) in males and 234 (4.68%) in the females. Furthermore, 1357 (31.08%) reported having hypertension in male and 1619 (32.37%) in the female. In multivariable-adjusted model, famine exposure and serum uric acid were associated with prevalence of hypertension independently in total populations [(1) Model four^d^, fatal exposed group vs non-exposed group: 1.25 (95% CI 1.03, 1.52); childhood-exposed group vs non-exposed group:1.60 (95% CI 1.37, 1.87); adolescence/adult exposed group vs non-exposed group: 2.87 (95% CI 2.44, 3.37), *P* for trend < 0.001; (2) Model four ^e^, high vs normal:1.73 (95% CI 1.44, 2.08)]. When stratified by sex, the results in both males and females were similar to those in the total population. In general, interaction analysis in the multivariable-adjusted model, compared with the combination of normal SUA level and no-exposed famine stage, all groups trended towards higher odds of prevalence of hypertension [the greatest increase in odds, adolescence/adult exposed stage and high SUA level in total participants: OR4.34; 95%CI 3.24, 5.81; *P* for interaction < 0.001]. When stratified by sex, the results in both males and females were also similar to those in the total population.

**Conclusion:**

Our data support a strongly positive individual and combined association of famine exposure and serum uric acid with hypertension in middle-aged and elderly Chinese.

## Background

Hypertension is a severe medical condition that significantly increases cardiovascular disease as well as other chronic diseases [1–3]. Hypertension risk factors [4–8] include stress, harmful use of alcohol, salt consumption, low intake of fruits and vegetables, being overweight or obese, saturated fat and trans fats, tobacco use, low diet in vitamin D, lack of physical activity, family history, aged 65 years or over and co-existing diseases. Though the etiology of hypertension is complex, it was known as one of the risk factors was high serum uric acid (SUA). Most mechanisms [9] supported that uric acid (UA) induced acute vasoconstriction by activation of renin-angiotensin system (RAS), followed by uric acid uptake into vascular smooth muscle cells leading to cellular proliferation and secondary arteriolosclerosis that results in chronic salt-sensitive hypertension. Thus, increased serum uric acid increases the risk of developing hypertension. In addition to known and probable risk factors for hypertension, early life mal-nutrition may also affect hypertension. Though the mechanisms are unclear, it is speculated that some fetal growth regulation factors might be most vulnerable to nutrient deficiencies, specifically during fetal and early postnatal life, dependent on the window of exposure [10].

It was hypothesized that early developmental adaptions in response to malnutrition in early life, which are key determinants of short-term survival, have adverse cardiovascular outcomes [11, 12]. Historical famine exposure has provided a unique and natural opportunity to test the hypothesis. Previous famine studies [10, 13–24] have provided pieces of evidence to support the association between famine exposure and increased risk of hypertension. Most studies [10, 13, 15, 17–24] found that exposure to famine in early life increases the risk of BP/hypertension in adulthood. Furthermore, exposure to famine has more deleterious effects on adult health for women than men [14, 16]. However, other studies [15, 25, 26] did not find a significant association between famine exposure and hypertension. Therefore, the association between famine exposure in early life and the risk of BP/hypertension in later life needs to be further studied. Moreover, results also indicated the malnutrition in early life were more strongly correlated with hyperuricemia in late life [27, 28]. It is not completed understood association and interaction analysis between famine exposure and serum uric acid and hypertension in the mid-age and older adult.

Given the limitations of previous studies, our research analyzed data from the China Health and Retirement Longitudinal Study (CHARLS) Wave1 and aimed to investigate the individual and combined association between famine exposure and serum uric acid and hypertension after adjustment for confounding variables.

## Methods

### Study design and setting

Data selected from the China Health and Retirement Longitudinal Study (CHARLS) Wave1 were analyzed in our research. The CHARLS is an ongoing nationally representative longitudinal study of middle-aged and elderly individuals in China that is conducted by the China Centre for Economic Research at Peking University [29] from 2011. In the 2011 CHARLS Wave1, at baseline, 17,707 individuals were recruited in the first visit. We selected individuals aged 45 years and older. After excluding participants who had missing values of the baseline characteristics and measurements, 9368 were included in our research. All data are openly published as microdata at http://charls.pku.edu.cn/index/zh-cn.html with no direct contact with all participants.

### Individuals

The individuals of the study were selected from the China Health and Retirement Longitudinal Study (CHARLS), Wave 1 (2011) [29]. The age of CHARLS involved 9368 individuals were [mean ± standard deviation age = 59.47 ± 9.31 years, and ranged from 45 to 90 years]. The mean and standard deviation of age were 60.23 ± 9.22 years (ranged from 45 to 90 years) in males and 58.80 ± 9.33 years (ranged from 45 to 90 years) in females.

### Baseline characteristics

Baseline variables including age, sex, education, marital status, living place, smoking status, alcohol consumption status, eating meals, social and leisure activities, the experience of a traumatic event, physical exercise habit were collected using a type of self-report method. Sex was categorized as male and female. Education was divided into four groups: illiterate, less than elementary school, high school, and above vocational school. Marital status was categorized into single and married. The living place was classed as rural and urban. Smoking status was defined as never smoker, former smoker, and current smoker. Alcohol consumption status was classed as no drinking, less than once a month, and more than once a month. Eating habit was divided into three groups: ≤ 2 meals per day, 3 meals per day, and ≥ 4 meals per day. Social and leisure activities, the experience of a traumatic event was categorized into “yes” and “no”. Physical exercise habit was defined as no exercise, less than regular physical exercises, and regular physical exercises. All variables were depending on our previous research studies [30–35].

### Measurements

Body measure index (BMI) was calculated based on the measured weight and height of the participants. Hypertension was defined as systolic blood pressure (SBP) of ≥ 140 mmHg and/or diastolic blood pressure (DBP) of ≥ 90 mmHg, the definition has been widely used in our previous studies[30, 32, 34, 35]. SUA was measured by the enzymatic colormetric test in the Youanmen Center for Clinical Laboratory at Capital Medical University. Hyperuricemia (HUA) was defined as SUA concentration of > 6 mg/dL in females and > 7 mg/dL in males [36].

### Exposure age and exposed stages

The famine period was between 1959 and 1962, famine exposure is set up on the birth year. Like the previous Chinese famine study [37], participants were categorized into four exposure groups: no-exposed stage (born between 1963-01-01 and 1966-12-31), fetal exposed stage (born between 1959-01-01 and 1962-12-31), childhood exposed stage (born between 1949-01-01 and 1958-12-31), adolescence/adult exposed stage (born between1921-01-01 and 1948-12-31).

### Statistical analysis

The data are presented as means and standard deviation (SD) unless indicated otherwise. Means and standard deviation (continuous data) were used to describe continuous variables (age, BMI), and number and percentage (categorical data) were used to assess the categorical variables (sex, education, marital status, living place, alcohol consumption status, smoking status, eating meals, social and leisure activities, the experience of a traumatic event, taking physical activity or exercise, SUA levels, famine exposure and hypertension categories). Differences between baseline characteristics (education, marital status, living place, social and leisure activities, the experience of a traumatic event, taking physical activity or exercise, hypertension) and categories of famine exposure stages/SUA levels were also evaluated using the chi-square test (categorical data). Between-group differences according to hypertension (hypertension, no-hypertension) were evaluated by the chi-square test (categorical data). Age and BMI between groups were used by t-test or F-test. For our research, logistic regression models were conducted to assess odds ratios (ORs) with accompanying 95% confidence intervals (95% CIs) as estimates of associations of SUA levels and exposure stages separately and in combination, with the prevalence of hypertension. Furthermore, the logistic regression models were employed to explore the linear trend *P-*value in subgroups. Famine exposure-SUA interaction analysis was examined by introducing the interaction term [famine exposure × SUA] into the confounder-adjusted logistic regression models. All statistical analyses were performed with SPSS software, version 25.0 (IBM SPSS, Armonk, NY, USA), and *P* < 5% was considered as a significant level.

## Results

Table 1 shows the basic characteristics of participants. A total of 9368 individuals were enrolled in the study, 4366 (46.61%) and 5002 (53.39%) were male and female, respectively. Among males, 459 (10.51%) had been exposed to the Chinese famine during the fetal stage, whereas 1760 (40.31%) and 1645 (37.68%) had been exposed to the famine during childhood and adolescence/adult stage, respectively. The distribution of physical exercises habit did not demonstrate a significantly statistical difference among the four birth groups. On the other hand, the differences were observed in the distribution of age, BMI, education, marital status, living place, cigarette smoking, alcohol consumption status, eating habit, social events, history of accidental injury, hypertension, and SUA levels. Among females, 635 (12.69%) had been exposed to the Chinese famine during the fetal stage, whereas 1988 (39.74%) and 1569 (31.37%) had been exposed to the famine during childhood and adolescence/adult stage, respectively. Furthermore, the distribution of living place and history of accidental injury did not demonstrate significantly statistical differences among the four birth groups. On the other hand, the difference was observed in the distribution of age, BMI, education, marital status, cigarette smoking, alcohol consumption status, eating habit, social events, physical exercises habit, hypertension, and SUA levels.

**Table 1:** Characteristics of participants in the cohort study by level of famine exposure (N = 9368)

Variables	Famine exposure in males N = 4366				χ2/F	*P*
	No-exposed	Fetal exposed	Childhood-exposed	Adolescence/adult-exposed		
N	502	459	1760	1645		
Age (years)	46.74 ± 1.07	50.28 ± 1.17	57.57 ± 2.83	69.97 ± 5.55	6738.564	< 0.001
BMI (kg/m^2^)	24.06 ± 3.59	23.72 ± 4.06	23.17 ± 3.53	22.27 ± 3.59	32.989	< 0.001
Education						
Illiterate	10 (1.99)	20 (4.36)	190 (10.8)	347 (21.09)	384.044	< 0.001
Less than elementary school	400 (79.68)	316 (68.85)	1346 (76.48)	1164 (70.76)		
High school	68 (13.55)	100 (21.79)	167 (9.49)	32 (1.95)		
Above vocational school	24 (4.78)	23 (5.01)	57 (3.24)	102 (6.2)		
Marital status						
Single	26 (5.18)	20 (4.36)	118 (6.7)	240 (14.59)	92.428	< 0.001
Married	476 (94.82)	439 (95.64)	1642 (93.3)	1405 (85.41)		
Living place						
Rural	317 (63.15)	293 (63.83)	1142 (64.89)	1129 (68.63)	8.829	0.032
Urban	185 (36.85)	166 (36.17)	618 (35.11)	516 (31.37)		
Smoking status						
No	133 (26.49)	101 (22)	393 (22.33)	450 (27.36)	66.125	< 0.001
Former smoke	66 (13.15)	48 (10.46)	286 (16.25)	347 (21.09)		
Current smoke	303 (60.36)	310 (67.54)	1081 (61.42)	848 (51.55)		
Alcohol habit						
No	168 (33.47)	164 (35.73)	723 (41.08)	855 (51.98)	85.718	< 0.001
Less than once a month	69 (13.75)	65 (14.16)	200 (11.36)	149 (9.06)		
More than once a month	265 (52.79)	230 (50.11)	837 (47.56)	641 (38.97)		
Eating habit						
≤ 2 meals per day	69 (13.75)	69 (15.03)	209 (11.88)	233 (14.16)	14.199	0.027
3 meals per day	428 (85.26)	388 (84.53)	1516 (86.14)	1394 (84.74)		
≥ 4 meals per day	5 (1)	2 (0.44)	35 (1.99)	18 (1.09)		
Social events						
No	195 (38.84)	193 (42.05)	894 (50.8)	864 (52.52)	39.989	< 0.001
Yes	307 (61.16)	266 (57.95)	866 (49.2)	781 (47.48)		
Experience of a traumatic event						
No	416 (82.87)	394 (85.84)	1529 (86.88)	1461 (88.81)	12.963	0.005
Yes	86 (17.13)	65 (14.16)	231 (13.13)	184 (11.19)		
Physical exercises habit						
No physical exercise	310 (61.75)	279 (60.78)	1087 (61.76)	1038 (63.1)	12.134	0.059
Less than regular physical exercises	96 (19.12)	103 (22.44)	348 (19.77)	272 (16.53)		
Regular physical exercises	96 (19.12)	77 (16.78)	325 (18.47)	335 (20.36)		
Hypertension						
No	390 (77.69)	333 (72.55)	1252 (71.14)	1034 (62.86)	53.108	< 0.001
Yes	112 (22.31)	126 (27.45)	508 (28.86)	611 (37.14)		
SUA levels						
Normal	474 (94.42)	426 (92.81)	1666 (94.66)	1510 (91.79)	12.438	0.006
High	28 (5.58)	33 (7.19)	94 (5.34)	135 (8.21)		

Variables	Famine exposure in females N = 5002				χ2/F	*P*
	No-exposed	Fetal exposed	Childhood-exposed	Adolescence/adult-exposed		
N	810	635	1988	1569		
Age (years)	46.76 ± 1.08	50.27 ± 1.17	57.56 ± 2.73	70.04 ± 5.87	8608.249	< 0.001
BMI (kg/m^2^)	24.75 ± 3.82	24.74 ± 3.96	24.11 ± 4.04	23.27 ± 4.3	43.834	< 0.001
Education						
Illiterate	137 (16.91)	173 (27.24)	896 (45.07)	921 (58.7)	618.607	< 0.001
Less than elementary school	587 (72.47)	348 (54.8)	981 (49.35)	610 (38.88)		
High school	57 (7.04)	103 (16.22)	87 (4.38)	11 (0.7)		
Above vocational school	29 (3.58)	11 (1.73)	24 (1.21)	27 (1.72)		
Marital status						
Single	23 (2.84)	35 (5.51)	184 (9.26)	501 (31.93)	547.311	< 0.001
Married	787 (97.16)	600 (94.49)	1804 (90.74)	1068 (68.07)		
Living place						
Rural	502 (61.98)	410 (64.57)	1257 (63.23)	1013 (64.56)	1.927	0.588
Urban	308 (38.02)	225 (35.43)	731 (36.77)	556 (35.44)		
Smoking status						
No	780 (96.3)	598 (94.17)	1838 (92.45)	1382 (88.08)	63.633	< 0.001
Former smoke	3 (0.37)	9 (1.42)	33 (1.66)	57 (3.63)		
Current smoke	27 (3.33)	28 (4.41)	117 (5.89)	130 (8.29)		
Alcohol habit						
No	709 (87.53)	547 (86.14)	1751 (88.08)	1378 (87.83)	20.027	0.003
Less than once a month	48 (5.93)	44 (6.93)	107 (5.38)	55 (3.51)		
More than once a month	53 (6.54)	44 (6.93)	130 (6.54)	136 (8.67)		
Eating habit						
≤ 2 meals per day	125 (15.43)	80 (12.6)	228 (11.47)	228 (14.53)	14.889	0.021
3 meals per day	680 (83.95)	547 (86.14)	1730 (87.02)	1318 (84)		
≥ 4 meals per day	5 (0.62)	8 (1.26)	30 (1.51)	23 (1.47)		
Social events						
No	366 (45.19)	271 (42.68)	1003 (50.45)	856 (54.56)	34.308	< 0.001
Yes	444 (54.81)	364 (57.32)	985 (49.55)	713 (45.44)		
Experience of a traumatic event						
No	771 (95.19)	589 (92.76)	1839 (92.51)	1455 (92.73)	6.943	0.074
Yes	39 (4.81)	46 (7.24)	149 (7.49)	114 (7.27)		
Physical exercises habit						
No physical exercise	478 (59.01)	365 (57.48)	1194 (60.06)	1020 (65.01)	16.762	0.010
Less than regular physical exercises	164 (20.25)	128 (20.16)	391 (19.67)	278 (17.72)		
Regular physical exercises	168 (20.74)	142 (22.36)	403 (20.27)	271 (17.27)		
Hypertension						
No	650 (80.25)	492 (77.48)	1410 (70.93)	831 (52.96)	251.082	< 0.001
Yes	160 (19.75)	143 (22.52)	578 (29.07)	738 (47.04)		
SUA levels						
Normal	792 (97.78)	615 (96.85)	1904 (95.77)	1457 (92.86)	36.492	< 0.001
High	18 (2.22)	20 (3.15)	84 (4.23)	112 (7.14)		

BMI, body mass index; SUA, serum uric acid

Table 2 shows the characteristics of study participants categorized by SUA levels. Of the participants, 290 (6.64%) reported having HUA in the male and 234 (4.68%) in the female. In males, significant differences were observed in age, BMI, living place, cigarette smoking, alcohol consumption status, physical exercises habit, famine exposed stages, and hypertension groups (*P* < 0.05) between participants with and without HUA. Regarding the females, significant differences were observed in age, BMI, marital status, living place, cigarette smoking, famine exposed stages, and hypertension groups (*P* < 0.05) between participants with and without HUA.

**Table 2:** Characteristics of participants in the cohort study by level of SUA levels (N = 9368)

Variables	SUA levels in male N = 4366		χ2/t	*P*	SUA levels in female N = 5002		χ2/t	*P*
	Normal	High			Normal	High		
N	4076	290			4768	234		
Age (years)	60.11 ± 9.17	61.90 ± 9.81	− 3.202	0.001	58.61 ± 9.27	62.65 ± 9.75	− 6.488	< 0.001
BMI (kg/m^2^)	22.64 ± 3.59	23.77 ± 3.74	− 4.937	0.000	23.66 ± 3.9	24.81 ± 4.45	− 5.112	< 0.001
Education								
Illiterate	531 (13.03)	36 (12.41)	2.741	0.433	2028 (42.53)	99 (42.31)	3.326	0.344
Less than elementary school	3019 (74.07)	207 (71.38)			2406 (50.46)	120 (51.28)		
High school	338 (8.29)	29 (10)			250 (5.24)	8 (3.42)		
Above vocational school	188 (4.61)	18 (6.21)			84 (1.76)	7 (2.99)		
Marital status								
Single	381 (9.35)	23 (7.93)	0.647	0.421	684 (14.35)	59 (25.21)	20.831	< 0.001
Married	3695 (90.65)	267 (92.07)			4084 (85.65)	175 (74.79)		
Living place								
Rural	2706 (66.39)	175 (60.34)	4.406	0.036	3054 (64.05)	128 (54.7)	8.427	0.004
Urban	1370 (33.61)	115 (39.66)			1714 (35.95)	106 (45.3)		
Smoking status								
No	997 (24.46)	80 (27.59)	8.849	0.012	4387 (92.01)	211 (90.17)	6.186	0.045
Former smoke	683 (16.76)	64 (22.07)			92 (1.93)	10 (4.27)		
Current smoke	2396 (58.78)	146 (50.34)			289 (6.06)	13 (5.56)		
Alcohol habit								
No	1792 (43.96)	118 (40.69)	12.946	0.002	4178 (87.63)	207 (88.46)	0.677	0.713
Less than once a month	466 (11.43)	17 (5.86)			241 (5.05)	13 (5.56)		
More than once a month	1818 (44.6)	155 (53.45)			349 (7.32)	14 (5.98)		
Eating habit								
≤ 2 meals per day	544 (13.35)	36 (12.41)	0.205	0.903	632 (13.26)	29 (12.39)	3.013	0.222
3 meals per day	3476 (85.28)	250 (86.21)			4076 (85.49)	199 (85.04)		
≥ 4 meals per day	56 (1.37)	4 (1.38)			60 (1.26)	6 (2.56)		
Social events								
No	2010 (49.31)	136 (46.9)	0.633	0.426	2380 (49.92)	116 (49.57)	0.011	0.918
Yes	2066 (50.69)	154 (53.1)			2388 (50.08)	118 (50.43)		
Experience of a traumatic event								
No	3550 (87.1)	250 (86.21)	0.189	0.663	4437 (93.06)	217 (92.74)	0.036	0.850
Yes	526 (12.9)	40 (13.79)			331 (6.94)	17 (7.26)		
Physical exercises habit								
No physical exercise	2517 (61.75)	197 (67.93)	6.367	0.041	2912 (61.07)	145 (61.97)	0.118	0.943
Less than regular physical exercises	780 (19.14)	39 (13.45)			918 (19.25)	43 (18.38)		
Regular physical exercises	779 (19.11)	54 (18.62)			938 (19.67)	46 (19.66)		
Famine exposure								
No-exposed	474 (11.63)	28 (9.66)	12.438	0.006	792 (16.61)	18 (7.69)	36.492	< 0.001
Fetal exposed	426 (10.45)	33 (11.38)			615 (12.9)	20 (8.55)		
Childhood-exposed	1666 (40.87)	94 (32.41)			1904 (39.93)	84 (35.90)		
Adolescence/adult-exposed	1510 (37.05)	135 (46.55)			1457 (30.56)	112 (47.86)		
Hypertension								
No	2851 (69.95)	158 (54.48)	30.222	0.000	3268 (68.54)	115 (49.15)	38.329	< 0.001
Yes	1225 (30.05)	132 (45.52)			1500 (31.46)	119 (50.85)		

BMI, body mass index; SUA, serum uric acid

Table 3 shows the characteristics of study participants categorized by blood pressure status. Of the participants, 1357 (31.08%) reported having hypertension in male and 1619 (32.37%) in the female. In males, significant differences were observed in age, BMI, education, marital status, living place, history of accidental injury, famine stages, and hypertension groups (*P* < 0.05) between participants with and without hypertension. In females, significant differences in distribution were observed between blood pressure status in the variables, including age, BMI, education, marital status, alcohol consumption status, famine stages, and SUA level groups.

**Table 3:** Characteristics of study participants of cross-sectional study categorized by blood pressure status (N = 9368)

Variables	Hypertension in male N = 4366		χ2/t	*P*	Hypertension in female N = 5002		χ2/t	*P*
	Without hypertension	Hypertension			Without hypertension	Hypertension		
N	3009	1357			3383	1619		
Age(years)	59.47 ± 9.02	61.92 ± 9.44	− 8.192	0.000	57.23 ± 8.59	62.08 ± 9.95	− 6.488	< 0.001
BMI (kg/m^2^)	22.92 ± 3.62	24.02 ± 4.24	− 9.481	0.000	23.97 ± 4.06	25.37 ± 4.99	− 9.329	< 0.001
Education								
Illiterate	376 (12.5)	191 (14.08)	8.267	0.041	1324 (39.14)	803 (49.6)	59.666	< 0.001
Less than elementary school	2230 (74.11)	996 (73.4)			1785 (52.76)	741 (45.77)		
High school	271 (9.01)	96 (7.07)			208 (6.15)	50 (3.09)		
Above vocational school	132 (4.39)	74 (5.45)			66 (1.95)	25 (1.54)		
Marital status								
Single	236 (7.84)	168 (12.38)	22.927	0.000	405 (11.97)	338 (20.88)	68.661	< 0.001
Married	2773 (92.16)	1189 (87.62)			2978 (88.03)	1281 (79.12)		
Living place								
Rural	2041 (67.83)	840 (61.9)	14.646	< 0.001	2175 (64.29)	1007 (62.2)	2.073	0.150
Urban	968 (32.17)	517 (38.1)			1208 (35.71)	612 (37.8)		
Smoking status								
No	742 (24.66)	335 (24.69)	0.503	0.778	3124 (92.34)	1474 (91.04)	2.502	0.286
Former smoke	507 (16.85)	240 (17.69)			65 (1.92)	37 (2.29)		
Current smoke	1760 (58.49)	782 (57.63)			194 (5.73)	108 (6.67)		
Alcohol habit								
No	1317 (43.77)	593 (43.7)	2.114	0.348	2935 (86.76)	1450 (89.56)	10.209	0.006
Less than once a month	346 (11.5)	137 (10.1)			193 (5.7)	61 (3.77)		
More than once a month	1346 (44.73)	627 (46.2)			255 (7.54)	108 (6.67)		
Eating habit								
≤ 2 meals per day	381 (12.66)	199 (14.66)	3.266	0.195	434 (12.83)	227 (14.02)	2.06	0.357
3 meals per day	2586 (85.94)	1140 (84.01)			2901 (85.75)	1374 (84.87)		
≥ 4 meals per day	42 (1.4)	18 (1.33)			48 (1.42)	18 (1.11)		
Social events								
No	1458 (48.45)	688 (50.7)	1.887	0.170	1698 (50.19)	798 (49.29)	0.357	0.550
Ye	1551 (51.55)	669 (49.3)			1685 (49.81)	821 (50.71)		
Experience of a traumatic event								
No	2595 (86.24)	1205 (88.8)	5.422	0.020	3138 (92.76)	1516 (93.64)	1.310	0.252
Yes	414 (13.76)	152 (11.2)			245 (7.24)	103 (6.36)		
Physical exercises habit								
No physical exercise	1868 (62.08)	846 (62.34)			2031 (60.04)	1026 (63.37)		
Less than regular physical exercises	575 (19.11)	244 (17.98)			662 (19.57)	299 (18.47)		
Regular physical exercises	566 (18.81)	267 (19.68)	1.013	0.603	690 (20.4)	294 (18.16)	5.470	0.065
Famine exposure								
No-exposed	390 (12.96)	112 (8.25)	53.108	< 0.001	650 (19.21)	160 (9.88)	251.082	< 0.001
Fetal exposed	333 (11.07)	126 (9.29)			492 (14.54)	143 (8.83)		
Childhood-exposed	1252 (41.61)	508 (37.44)			1410 (41.68)	578 (35.7)		
Adolescence/adult-exposed	1034 (34.36)	611 (45.03)			831 (24.56)	738 (45.58)		
SUA levels								
Normal	2851 (94.75)	1225 (90.27)	30.222	< 0.001	3268 (96.6)	1500 (92.65)	38.329	< 0.001
High	158 (5.25)	132 (9.73)			115 (3.4)	119 (7.35)		

BMI, body mass index; SUA, serum uric acid

Table 4 shows the separate associations of famine exposure, SUA levels with the prevalence of hypertension. Firstly, after controlling for confounding factors including age, education, marital status, living place, smoking status, alcohol consumption status, eating meals, social and leisure activities, the experience of a traumatic event, taking physical activity or exercise, BMI, and famine exposure in a multivariable logistic regression model four, higher odds of prevalence of hypertension in the total population were observed with increasing levels of SUA [high vs normal:1.73 (95% CI 1.44, 2.08) independently of famine stages only. When stratified by sex, the results of model four in both males and females were similar to those in the total population. Secondly, after controlling for confounding factors including age, education, marital status, living place, smoking status, alcohol consumption status, eating meals, social and leisure activities, the experience of a traumatic event, taking physical activity or exercise, BMI, and SUA levels in a multivariable logistic regression model four, higher odds of prevalence of hypertension in the total population were observed with famine exposed stages [fatal exposed group vs non-exposed group: 1.25 (95% CI 1.03, 1.52); childhood-exposed group vs non-exposed group:1.60 (95% CI 1.37, 1.87); adolescence/adult exposed group vs non-exposed group: 2.87 (95% CI 2.44, 3.37), *P* for trend < 0.001] independently of SUA levels only. When stratified by sex, the results of model four in both males and females were similar to those in the total population.

**Table 4:** Separate associations of famine exposure, SUA levels with prevalence of hypertension (N = 9368)

Variables	Male (OR and 95% CI for hypertension)				Female (OR and 95% CI for hypertension)			
Famine exposure	Model one^a^	Model two^b^	Model three^c^	Model four^d^	Model one^a^	Model two^b^	Model three^c^	Model four^d^
No-exposed	1.00 (reference)	1.00 (reference)	1.00 (reference)	1.00 (reference)	1.00 (reference)	1.00 (reference)	1.00 (reference)	1.00 (reference)
Fetal exposed	1.32 (0.98, 1.77)	1.32 (0.98, 1.77)	1.30 (0.97, 1.75)	1.35 (1.00, 1.82)	1.18 (0.92, 1.52)	1.16 (0.90, 1.49)	1.17 (0.91, 1.51)	1.16 (0.90, 1.51)
Childhood-exposed	1.41 (1.12, 1.79)	1.42 (1.12, 1.79)	1.41 (1.11, 1.79)	1.54 (1.21, 1.96)	1.67 (1.37, 2.03)	1.51 (1.24, 1.85)	1.54 (1.25, 1.88)	1.62 (1.32, 1.99)
Adolescence/adult-exposed	2.06 (1.63, 2.60)	1.98 (1.56, 2.51)	1.99 (1.57, 2.52)	2.38 (1.86, 3.04)	3.61 (2.96, 4.40)	2.98 (2.41, 3.68)	3.04 (2.45, 3.76)	3.46 (2.78, 4.30)
*P* for trend	< 0.001	< 0.001	< 0.001		< 0.001	< 0.001	< 0.001	< 0.001
SUA levels	Model one^a^	Model two^f^	Model three^g^	Model four^e^	Model one^a^	Model two^f^	Model three^g^	Model four^e^
Normal	1.00 (reference)	1.00 (reference)	1.00 (reference)	1.00 (reference)	1.00 (reference)	1.00 (reference)	1.00 (reference)	1.00 (reference)
High	1.94 (1.53, 2.47)	1.89 (1.48, 2.42)	1.90 (1.48, 2.42)	1.74 (1.36, 2.23)	2.25 (1.73, 2.93)	1.93 (1.47, 2.53)	1.93 (1.47, 2.54)	1.72 (1.31, 2.27)

Variables	Total (OR and 95% CI for hypertension)			
Famine exposure	Model one^a^	Model two^b^	Model three^c^	Model four^d^
No-exposed	1.00 (reference)	1.00 (reference)	1.00 (reference)	1.00 (reference)
Fetal exposed	1.25 (1.03, 1.51)	1.24 (1.02, 1.50)	1.24 (1.02, 1.51)	1.25 (1.03, 1.52)
Childhood-exposed	1.56 (1.34, 1.81)	1.48 (1.27, 1.73)	1.50 (1.29, 1.75)	1.60 (1.37, 1.87)
Adolescence/adult-exposed	2.77 (2.38, 3.22)	2.44 (2.09, 2.84)	2.46 (2.10, 2.87)	2.87 (2.44, 3.37)
*P* for trend	< 0.001	< 0.001	< 0.001	< 0.001
SUA levels	Model one^a^	Model two^f^	Model three^g^	Model four^e^
Normal	1.00 (reference)	1.00 (reference)	1.00 (reference)	1.00 (reference)
High	2.06 (1.73, 2.46)	1.91 (1.59, 2.29)	1.91 (1.60, 2.29)	1.73 (1.44, 2.08)

BMI, body mass index; CI, confidence interval; OR, odds ratios; SUA, serum uric acid; SBP, systolic blood pressure

(1) In model one, ^a^Unadjusted, age-adjusted by design;

(2) In model two: ^b^Adjusted for age, education, marital status, living place, and SUA; ^f^ Adjusted for age, education, marital status, living place, and famine exposure

(3) In model three: ^c^Adjusted for age, education, marital status, living place, smoking status, alcohol consumption status, eating habit, social and leisure activities, experience of a traumatic event, taking physical activity or exercise, and SUA; ^g^Adjusted for age, education, marital status, living place, smoking status, alcohol consumption status, eating habit, social and leisure activities, experience of a traumatic event, taking physical activity or exercise, and famine exposure

(4) In model four: ^d^Adjusted for age, education, marital status, living place, smoking status, alcohol consumption status, eating habit, social and leisure activities, experience of a traumatic event, taking physical activity or exercise, BMI, and SUA; ^e^Adjusted for age, education, marital status, living place, smoking status, alcohol consumption status, eating habit, social and leisure activities, experience of a traumatic event, taking physical activity or exercise, BMI, and famine exposure

Table 5 shows the combined associations of SUA levels and famine exposure with the prevalence of hypertension. Compared with the combination of normal SUA level and no-exposed famine stage, all groups trended towards higher odds of prevalence of hypertension; Furthermore, in multivariable model one, the greatest increase in odds was observed for the adolescence/adult exposed stage and high SUA level combination (adolescence/adult exposed stage and HUA in total participants: OR 4.37; 95%CI 3.28,5.81). And similarly, in multivariable-adjusted model two, the highest odds of prevalence of hypertension were observed for the adolescence/adult exposed stage and HUA combination (adolescence/adult exposed stage and high SUA in total participants: OR 3.94; 95%CI 2.96, 5.26). Additionally, in multivariable-adjusted model three, the highest odds of prevalence of hypertension were observed for the adolescence/adult exposed stage and high SUA combination (adolescence/adult exposed stage and HUA in total participants: OR3.99; 95%CI 2.99, 5.32). In multivariable-adjusted model four, the highest odds of prevalence of hypertension were observed for the adolescence/adult exposed stage and HUA combination (adolescence/adult exposed stage and high SUA level in total participants: OR4.34; 95%CI 3.24, 5.81). Finally, combined associations of high SUA levels and famine exposure with the prevalence of hypertension were observed in the total participant (*P*-interaction < 0.001). When stratified by sex, the results of the model in both males and females were similar to those in the total population.

**Table 5:** Combined associations of SUA levels and famine exposure with prevalence of hypertension (N = 9368)

Famine exposure	Prevalence of hypertension odds ratio (95%*CI*)							
	Model one^a^		Model two^b^		Model three^c^		Model four^d^	
	SUA levels		SUA levels		SUA levels		SUA levels	
Male	Normal	High	Normal	High	Normal	High	Normal	High
No-exposed	1.00 (reference)	1.81 (0.79, 4.14)	1.00 (reference)	1.79 (0.78, 4.12)	1.00 (reference)	1.76 (0.76, 4.06)	1.00 (reference)	1.65 (0.7, 3.88)
Fetal exposed	1.29 (0.95, 1.75)	2.66 (1.29, 5.49)	1.30 (0.95, 1.76)	2.75 (1.33, 5.68)	1.28 (0.94, 1.75)	2.69 (1.30, 5.58)	1.35 (0.99, 1.84)	2.22 (1.05, 4.7)
Childhood-exposed	1.39 (1.09, 1.77)	3.32 (2.09, 5.25)	1.39 (1.09, 1.78)	3.26 (2.06, 5.18)	1.38 (1.08, 1.77)	3.29 (2.07, 5.23)	1.51 (1.17, 1.93)	3.35 (2.1, 5.36)
Adolescence/adult-exposed	2.05 (1.61, 2.61)	3.26 (2.18, 4.87)	2.00 (1.57, 2.56)	3.17 (2.12, 4.75)	2.00 (1.57, 2.57)	3.17 (2.11, 4.75)	2.40 (1.86, 3.09)	3.56 (2.36, 5.38)
*P* for trend	< 0.001	< 0.001	< 0.001	< 0.001	< 0.001	< 0.001	< 0.001	< 0.001
*P*-interaction	< 0.001		< 0.001		< 0.001		< 0.001	

Female	Normal	High	Normal	High	Normal	High	Normal	High
No-exposed	1.00 (reference)	2.07 (0.77, 5.61)	1.00 (reference)	2.07 (0.76, 5.62)	1.00 (reference)	1.98 (0.73, 5.38)	1.00 (reference)	1.86 (0.68, 5.08)
Fetal exposed	1.18 (0.91, 1.52)	2.23 (0.88, 5.69)	1.16 (0.90, 1.51)	2.23 (0.87, 5.70)	1.17 (0.90, 1.52)	2.18 (0.85, 5.59)	1.17 (0.9, 1.52)	1.88 (0.72, 4.91)
Childhood-exposed	1.63 (1.33, 2.00)	3.77 (2.37, 5.98)	1.50 (1.22, 1.85)	3.41 (2.14, 5.43)	1.52 (1.24, 1.87)	3.50 (2.19, 5.58)	1.62 (1.31, 1.99)	3.09 (1.91, 4.98)
Adolescence/adult-exposed	3.55 (2.89, 4.35)	5.94 (3.92, 9.01)	3.01 (2.43, 3.74)	4.99 (3.27, 7.62)	3.07 (2.47, 3.81)	5.12 (3.35, 7.82)	3.49 (2.8, 4.36)	5.41 (3.52, 8.34)
*P* for trend	< 0.001	< 0.001	< 0.001	< 0.001	< 0.001	< 0.001	< 0.001	< 0.001
*P*-interaction	< 0.001		< 0.001		< 0.001		< 0.001	

Total	Normal	High	Normal	High	Normal	High	Normal	High
No-exposed	1.00 (reference)	1.96 (1.04, 3.71)	1.00 (reference)	1.94 (1.03, 3.67)	1.00 (reference)	1.93 (1.02, 3.65)	1.00 (reference)	1.80 (0.95, 3.45)
Fetal exposed	1.23 (1.01, 1.5)	2.58 (1.46, 4.55)	1.23 (1.01, 1.50)	2.66 (1.50, 4.69)	1.23 (1.01, 1.50)	2.69 (1.52, 4.75)	1.25 (1.03, 1.53)	2.23 (1.24, 4.01)
Childhood-exposed	1.53 (1.31, 1.79)	3.59 (2.60, 4.97)	1.47 (1.26, 1.72)	3.37 (2.43, 4.67)	1.49 (1.27, 1.74)	3.43 (2.47, 4.75)	1.59 (1.36, 1.87)	3.25 (2.33, 4.53)
Adolescence/adult-exposed	2.74 (2.35, 3.2)	4.37 (3.28, 5.81)	2.47 (2.11, 2.90)	3.94 (2.96, 5.26)	2.49 (2.12, 2.93)	3.99 (2.99, 5.32)	2.90 (2.47, 3.42)	4.34 (3.24, 5.81)
*P* for trend	< 0.001	< 0.001	< 0.001	< 0.001	< 0.001	< 0.001	< 0.001	< 0.001
*P*-interaction	< 0.001		< 0.001		< 0.001		< 0.001	

BMI, body mass index; CI, confidence interval; OR, odds ratio; SUA, serum uric acid; SBP, systolic blood pressure

(1) In model one: ^a^Unadjusted; age-adjusted by design

(2) In model two: ^b^Adjusted for age, education, marital status, living place

(3) In model three: ^c^Adjusted for age, education, marital status, living place, smoking status, alcohol consumption status, eating habit, social and leisure activities, experience of a traumatic event, taking physical activity or exercise;

(4) In model four: ^d^Adjusted for age, education, marital status, living place, smoking status, alcohol consumption status, eating habit, social and leisure activities, experience of a traumatic event, taking physical activity or exercise, and BMI

## Discussion

Our research aimed to explore the individual and combined association between famine exposure and serum uric acid and hypertension in mid-aged and older adults. Interestingly, our study found that the individuals exposed to famine in early life had an increased risk of hypertension in adult. After adjustment for observed confounders, including age, education, marital status, living place, smoking status, alcohol consumption status, eating meals, social and leisure activities, the experience of a traumatic event, taking physical activity or exercise, BMI, and SUA level, the associations still existed both in males and females. Additionally, the study showed that there were linear trends in the associations of SUA with hypertension. After adjustment for observed confounders, including age, education, marital status, living place, smoking status, alcohol consumption status, eating meals, social and leisure activities, the experience of a traumatic event, taking physical activity or exercise, BMI, and famine exposure, the associations still existed both in males and females. In general, our data support a strongly positive individual and combined association of famine exposure and SUA levels with hypertension in middle-aged and elderly Chinese. The outcomes of these individuals stratified by sex were examined. The results in both males and females were also similar to those in the total population.

The Chinese famine ranged from the late1950s to the early 1960s, caused over 30 million excess deaths in most areas [38]. Most studies have reported the associations of famine exposure during early life with hypertension in adults, but no consistent associations were observed. Therefore, this research attempted to examine the individual and combined association between famine exposure and serum uric acid and hypertension based on a population-based cross-sectional study from CHARLS. Our data support a strongly positive combined association of famine exposure and serum uric acid with hypertension in middle-aged and elderly Chinese. Both nutrition intervention for exposure to the famine in early life and serum uric acid reduction in later life may be required to substantially reduce the prevalence of hypertension.

As the worst famine, the survivors might be healthier than the weak members were kicked out, a common finding that is in line with Darwin’ s theory of survival of the fittest [39]. In this case, the participants exposed to famine in early life should decrease the risk of hypertension in adults. This was not observed in our research. When facing the later “rich” environment, the risk of hypertension may be increased. The outcomes are partly in line with previous studies. Although the Dutch famine and the Leningrad siege study [40–42] found that early-life exposure to famine was not associated with hypertension, most studies [10, 13, 14, 16–24] in China indicated that exposure to famine in early life increased the risk of hypertension. However, this association did not exist between the Chinese famine and hypertension risk in Chongqing [25]. Such discrepancies between those studies may be a result of methodological differences in definitions of famine exposure groups and the different sample selection effect. Additionally, these studies have been criticized for not being adjusted the effect of age. To control the potential age confounding, we categorized the famine exposure into four exposure groups [no-exposed stage (born between1963 and 1966), fetal exposed stage (born between1959 and 1962), childhood exposed stage (born between 1949 and 1958), adolescence/adult exposed stage (born between1921 and 1948)] based on the birth year and we also combined the no-exposure as the reference group to identify the effect of the fetal exposed stage, childhood exposed stage, adolescence/adult exposed stage. Our results suggested that early famine exposure was associated with an increased risk of hypertension. The sex difference of early life famine exposure and hypertension were common in several studies [16, 18]. Furthermore, exposure to famine during early life exerted more deleterious association on women than men. This could be explained by the fact the women may suffer more than men during the famine because of the dominance of a patriarchal mentality in China [43]. The main potential mechanisms of the relationship between famine exposure in early life and the increased risk of hypertension in later life were still not fully understood. Animal models [44, 45] have proved that undernutrition in early life could lead to hypertension in later life. In addition, epigenetic might play a part role in the relationship between famine exposure in early life and hypertension in adults [46, 47]. Though the potential mechanisms are unclear, it is speculated that some fetal growth regulation factors might be most vulnerable to nutrient deficiencies, specifically during fetal and early postnatal life, dependent on the window of exposure [10].

Although previous studies [48–55] have estimated the association between serum uric acid level and blood pressure/hypertension. However, the results are not consistent. Y. Kansui, T. Ohtsubo, K. Goto, et al.[48]found that both systolic and diastolic blood pressures were significantly correlated with serum uric acid among Japanese male workers aged 18–64 years. Lyngdoh et al. [49] found that adiposity substantially decreased the association between SUA and BP in adults, and BP was independently associated with SUA in females. Kawamoto et al. [50] found that serum uric acid level significantly associated with both systolic blood pressure (SBP) and diastolic blood pressure (DBP) in females aged < 55 years but not in those aged ≥ 55 years. Irijanto et al. [53]found that community-dwelling Japanese men with a BMI ≥ 21.0 kg/m^2^, serum uric acid level was positively correlated with SBP and DBP, but negatively associated with SBP and DBP in those with a BMI of ≥ 21.0 kg/m^2^. Lin et al. [55] found that serum UA levels are significantly correlated to BP in Taiwanese adolescents aged 14–19 years. Khanum et al. [56] found that the relationship between elevated SUA level and incident hypertension was observed among individuals aged < 55 years, but not observed among participants aged ≥ 55 years. Cao et al. [57] found that the positive relationship between elevated SUA level and hypertension was proved in a Chinese population. Several longitudinal studies [58–61] found that the serum uric acid level was positively related with the risk of incident hypertension independently. Similarly, other cross-sectional [62–64] also found that hyperuricemia was significantly related with the risk of hypertension. The difference between those studies may due to the different confounding variables by controlling, the different populations, and different sampling methods selection. Several hypotheses partly explain the association between SUA level and high blood pressure/hypertension. One of the possible mechanism might be uric acid deposition on the blood vessels walls activates the renin-angiotensin system, suppress the liberate of carbon monoxide, enhance inflammation, and leads to vasoconstriction later [62]. Another possibility involving oxidative stress and endothelial dysfunction related with high SUA level may contribute to elevated blood pressure [65, 66].

There were so many studies that had explored the association analysis between famine exposure/ obesity parameters and BP/hypertension, and only two studies that explored the combined association between famine exposure and obesity parameters and hypertension, but no study was aimed to investigate the combined association of famine exposure and serum uric acid with hypertension after adjustment for confounding variables. Yu et al.[20] found that interactions between famine and obesity on hypertension prevalence risk were not observed. In contrast, Li et al. [13] reported that a stronger interaction between obesity and famine exposure concerning BP among individuals who were exposed to famine during fetal life and had a western dietary pattern in adults was observed. Two studies [27, 28] also found famine exposure was associated with an increased risk of hyperuricemia in adulthood. Interestingly, our data support a strongly positive combined association of famine exposure and serum uric acid with hypertension in middle-aged and elderly Chinese.

There were several limitations to the study. First, selection bias was to be considered: famine may weed out the frail members of the population and leave the healthier ones. Second, famine exposure for each individual was unknown. Third, not all families were equally affected by famine exposure. Fourth, the data was collected in 2011, and more recent studies are needed to identify the associations. However, the results provided large data that could be explored further in the combined association of famine exposure and serum uric acid with hypertension. Moreover, a significant strength of the study is the large sample of 9368 middle-aged and older Chinese. Another strength is the analytical method the controlled the potential confounders.

## Conclusions

Our data support a positive individual and combined association of famine exposure and serum uric acid with hypertension in middle-aged and elderly Chinese. Both nutrition intervention for exposure to the famine in early life and serum uric acid reduction in later life may be required to substantially reduce the risk of hypertension.

## Data Availability

The datasets generated and/or analyzed during the current study are publicly available in the http://charls.pku.edu.cn/index.html repository.

## Abbreviations

BMI: Body mass index
CHARLS: China Health and Retirement Longitudinal Study
CI: Confidence interval
DBP: Diastolic blood pressure
NSFC: The National Natural Science Foundation of China
UA: Uric acid
RAS: Renin-angiotensin system
SD: Standard deviation
NIA: National Institute on Aging
OR: Odds ratios
SUA: Serum uric acid
HUA: High serum uric acid
UA: Uric acid
SBP: Systolic blood pressure
